# Emergency Department Approach to Testicular Torsion: Two Illustrative Cases

**DOI:** 10.7759/cureus.5967

**Published:** 2019-10-22

**Authors:** Sherwin Z Thomas, Vanessa I Diaz, Javier Rosario, Vibhav Kanyadan, Latha Ganti

**Affiliations:** 1 Emergency Medicine, University of Central Florida College of Medicine, Orlando, USA; 2 Emergency Medicine, University of Central Florida / Osceola Regional Medical Center, Orlando, USA; 3 Miscellaneous, Wheeler High School, Marietta, USA; 4 Emergency Medicine, Envision Physician Services, Orlando, USA

**Keywords:** testicular torsion

## Abstract

We present two cases of young men with spontaneous nontraumatic testicular pain. While the differential diagnosis for scrotal or testicular pain can include less urgent causes, such as epididymitis, hydrocele, referred pain, idiopathic scrotal edema, and inguinal hernia, for example, the most feared etiology for acute scrotal pain is testicular torsion. The fact that a testicle can torse and detorse is also a confounding factor. In this case review, we explore factors affecting the timely diagnosis, management, and outcomes of acute testicular pain. Prompt diagnosis is imperative in order to salvage a torsed testicle.

## Introduction

Acute scrotal pain is not an uncommon presentation to the emergency department (ED), accounting for approximately 0.5% of ED visits [1]. Each half of the scrotum contains the testis, epididymis, spermatic cord, and cremaster muscle. Testicular torsion occurs when the testicle twists around the spermatic cord, resulting in blood flow to the testicle being compromised [1]. This is a urologic emergency that affects one in 4000 males younger than 25 years annually [2] and results in an orchiectomy 42% of the time in those undergoing surgery for testicular torsion [3-4]. Torsion of testicular appendages, usually presenting in children in the age group of seven to 13 years, accounts for 24% to 46% of acute scrotal presentation [3]. Prompt recognition and treatment are critical for testicular salvage; thus, testicular torsion needs to be ruled out in all patients who present with acute scrotal pain [4-5].

The significant pathological change in testicular torsion is ischemia. Testicular rotation within the scrotum and rotation of the spermatic cord can cause inadequate blood fusion of the testicle to the scrotal wall and results in ischemia [1,5]. The rotation degree of testicular torsion is directly correlated with the possibility of salvage after torsion and time to ischemic necrosis [2].

The clinical presentation of testicular torsion is usually acute-onset, intense, unilateral scrotal pain with a similar previous history. Symptoms may also include nausea and vomiting that are secondary to pain. A "high-riding" testis is the hallmark of testicular torsion, which may be due to the shortening of the spermatic cord. A normal cremasteric reflex is rarely observed in patients with testicular torsion [6]. Additionally, the testis may have an abnormal (e.g., transverse) position in the scrotum. Workup should include urinalysis and scrotal ultrasound. The finding of hematuria or leukocytosis in urinalysis is more typical of epididymo-orchitis than testicular torsion. The reduction or absence of testicular blood flow assessed by Doppler ultrasonography is compelling for testicular torsion, although false interpretations may occur in young children or neonates with small blood vessels.

It is generally believed that the time window for possible salvage and survival of a torsed testicle is six to eight hours. However, the survival of torsed testicles with or without subsequent atrophy is known to occur outside that critical time window. The literature review shows that testicular salvage in the first six hours is 90%-100%, from six to 12 hours survival is 20%-50%, and beyond 12 hours survival is 11% [7]. It is important to note that the duration of symptoms should not be used to decide on management since the testicle can torse and then detorse, which would “reset” the clock [8].

This case report discusses two different cases and explores factors affecting the timely diagnosis, management, and outcomes of testicular torsion.

## Case presentation

Case of patient 1

**Figure 1:**
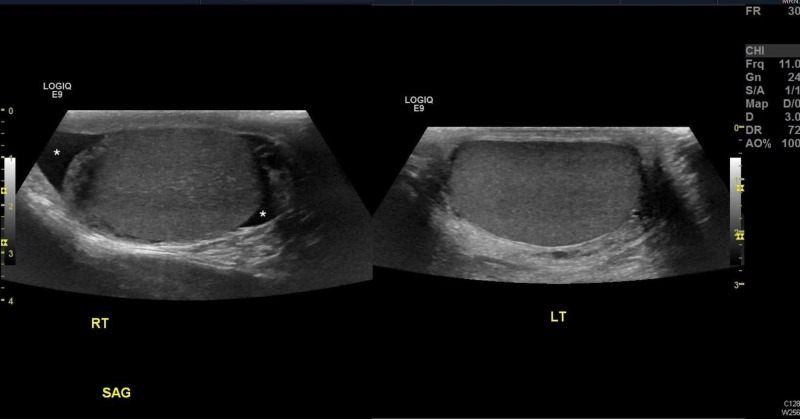
Note that the right testicle (RT) appears slightly hypoechoic when compared to the left testicle (LT). Hydrocele (asterisks) is noted on the right testicle, likely due to inflammatory changes being caused by the testicular torsion. LOGIC E9 = Images obtained on General Electric Ultrasonography machine General Electric, Massachusetts, United States SAG: sagittal view

**Figure 2:**
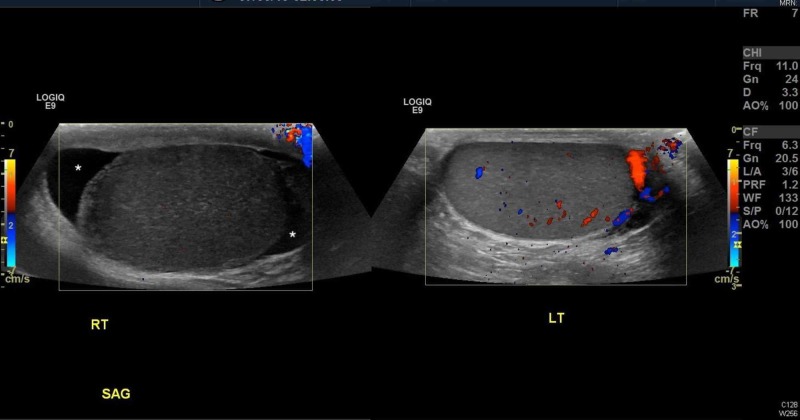
Comparison of arterial and venous flow between the two testicles. Note the lack of flow in the right testicle (RT) when compared to the left testicle (LT). Asterisks show hydrocele.

An 18-year-old male with no past medical or surgical history presented to a free-standing clinic with the chief complaint of right testicular pain. Symptoms started one hour prior to arrival, was exacerbated by palpation, relieved by nothing, was sharp and non-radiating, rated 10/10, and was constant, with no associated symptoms. The patient was sitting at home playing video games and drinking a few beers when he got up and started having intense right testicular pain. He was asymptomatic before that time and had no history of trauma, minor or otherwise. He came straight to the emergency department after symptoms developed. A scrotal ultrasound was ordered immediately. Ultrasonography (Figures 1-2) demonstrated the right testicle to have no sonographic demonstration of color flow, enlargement of the right epididymis, and hydrocoele. The left testicle demonstrates arterial and venous signals on color flow, a small left epididymal cyst, and a small left hydrocele.

The transfer center was called and the patient was sent to our facility for urology consultation. The patient arrived at our facility four hours after symptoms started and urology was consulted immediately. The review of systems was positive only for right testicular pain. Physical examination was abnormal only for a hard, high-riding right testicle with swelling and absent cremasteric reflex. The patient was taken straight to the operating room where the right testicle was found to be dark and rotated 720 degrees. After detorsion, the testicle started taking back some color so a bilateral orchidopexy was performed and the patient was discharged home the next day with an improvement of symptoms.

Case of patient 2

**Figure 3:**
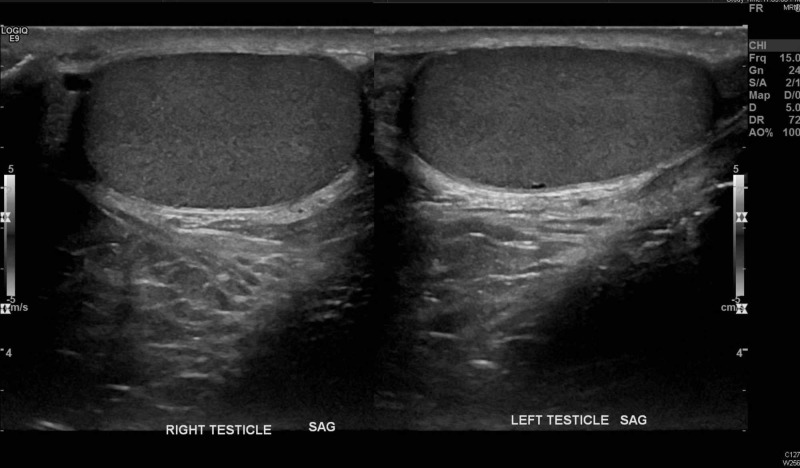
Bilateral testes side-by-side comparison showing isoechoic texture within the two testes likely representing equal flow SAG: sagittal view

**Figure 4:**
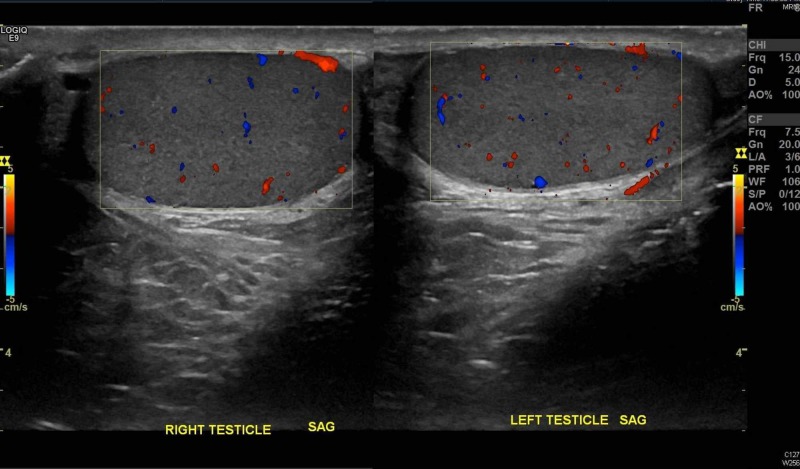
Doppler imaging showing bilateral arterial and venous flow within testes SAG: sagittal view

A 24-year-old male with no past medical or surgical history presented to our hospital with the chief complaint of right testicular pain. Symptoms started 24 hours prior to arrival, exacerbated by movement, relieved by nothing, sharp, radiating to the right flank, rated 5/10, intermittent, with no associated symptoms. The patient was sitting at home the previous night playing video games and drinking a few beers when he got up and started having right testicular pain and right flank pain. The patient was asymptomatic before that time and had no history of trauma, minor or otherwise. He decided to try and “sleep it off.” After 24 hours of worsening right testicular pain, he came to the emergency department. The review of systems was positive only for right flank and testicular pain. The exam was abnormal only for mild right costovertebral angle tenderness and mild right testicular tenderness. Laboratory investigations, including complete blood count, electrolytes, liver function tests, urinalysis, and chlamydia/gonorrhea polymerase chain reaction (PCR) testing, were unremarkable. An abdominal and pelvic computed tomography (CT) scan was also negative. An ultrasound was ordered immediately. Ultrasonography (Figures 3-4) demonstrated no significant hydrocele or varicocele. Both testicles had a homogeneous echotexture without evidence of mass or calcification. Color Doppler flow was seen on both testes.

After providing strict return precautions as well as outpatient urology follow-up, the patient was discharged home with an improvement of symptoms.

## Discussion

Testicular pain can encompass a surprisingly large number of differential diagnoses. For the emergency physician, the emergent culprits to rule out are infection/abscess (the most severe being Fournier's gangrene), incarcerated hernia, rupture, and torsion. The diagnosis of testicular torsion is indeed one of the most important to rule out since it is among the most common diagnoses of emergency physician medical malpractice cases in this age group [2]. Testicular torsion is a difficult diagnosis to rule out based on history and physical examination.

Patients can have testicular pain alone or in conjunction with non-specific signs such as nausea or vomiting. The physical exam is, therefore, vital in any situation when a patient endorses even a hint of testicular pain. Classic physical exam findings often taught to medical students are swollen, high-riding testicles with a transverse lie and absent cremasteric reflex. This last point is often used on examination questions to rule out testicular torsion. However, a normal cremasteric reflex is found to be present in an ever-increasing amount of confirmed testicular torsion cases [1]. So what is an emergency physician to do?

One scoring tool that can aid the emergency physician is the TWIST (testicular workup for ischemia and suspected torsion) score [3] (Table 1). Developed from a prospective study evaluating 338 children in a single institution, the score was created to aid in the initial diagnostic decision-making of testicular torsion. Multiple review studies since have confirmed a score of >5 has a positive predictive value (PPV) of 100% (thus, consult urology, there is no need for an ultrasound) and a score of <2 has an NPV of 100% (thus, unlikely to be torsion; there is no need for an ultrasound). Of course, the obvious limitation of this score is that it was derived utilizing a pediatric population rather than an adult population. Nevertheless, it may still be useful to adult emergency physicians to expedite diagnosis. In the case of patient 1 for instance, his TWIST score at the free-standing emergency department would have been 6, which, in a pediatric patient, would indicate urgent urology consult without the need for ultrasound. Meaning, patient 1 could have been transferred to our center 90 minutes sooner than he was. Patient 2’s TWIST score, on the other hand, was 0, meaning he, theoretically, did not even need an ultrasound.

**Table 1:** TWIST score for testicular torsion TWIST: testicular workup for ischemia and suspected torsion

Clinical feature	# points
Presence of testicular swelling	2
Absence of cremasteric reflex	1
Presence of high riding testicle	1
Presence of nausea/vomiting	1
Score interpretation	Recommended course of action
Score 6-7, high risk	Immediate urological consultation for surgical exploration
Score 1-5 intermediate risk	Obtain scrotal ultrasound, consider alternative diagnoses
Score 0, low risk	Unlikely to be torsion, consider other diagnoses

**Figure 5:**
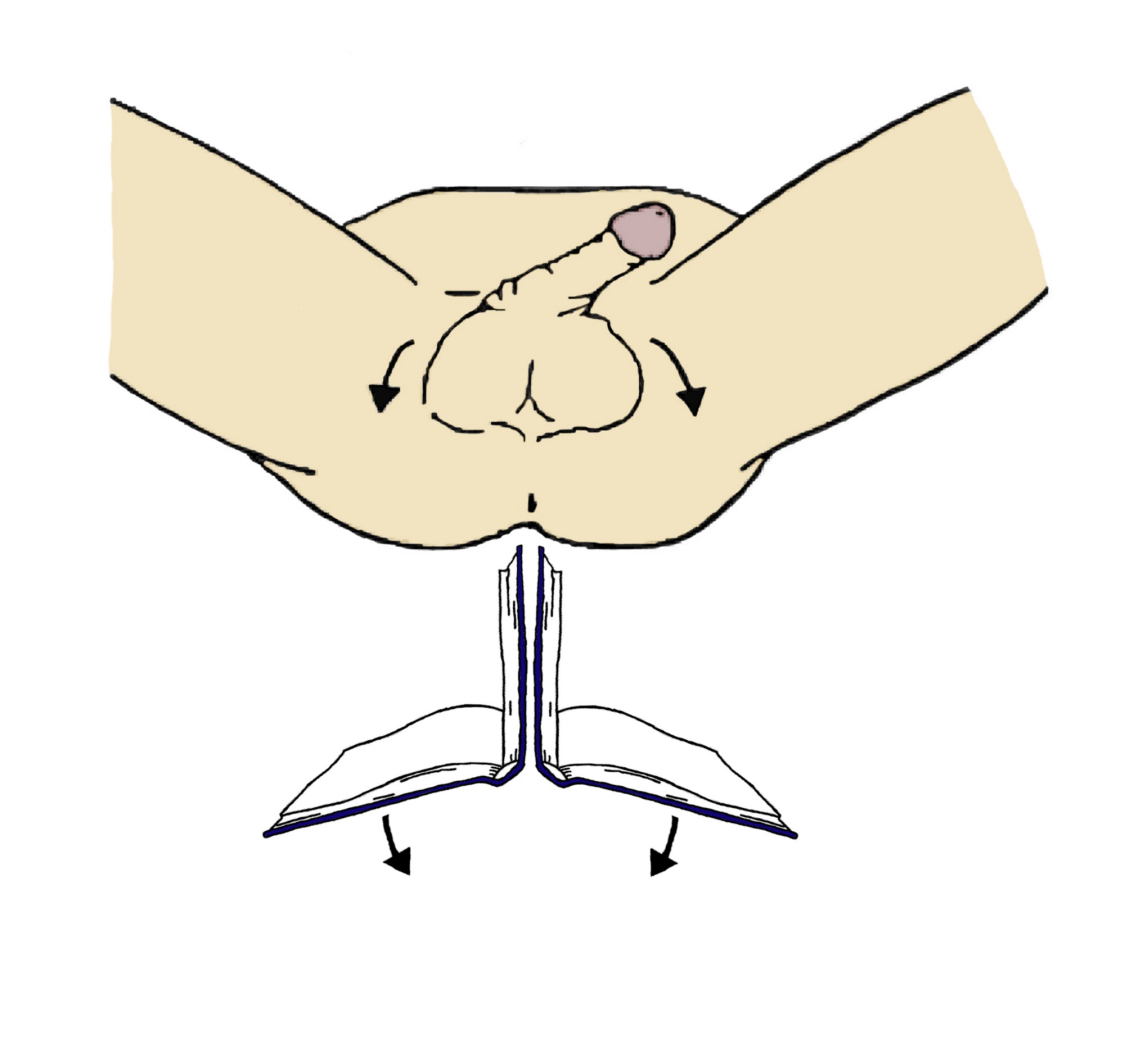
Manual detorsion maneuver for testicular torsion. With the physician facing the patient, the right testis is rotated clockwise while the left is rotated counterclockwise. This is referred to as the "open book" maneuver, as the movement is akin to opening a book. (Artwork by Dr. Amanda Webb)

In addition to taking the TWIST score into consideration (noting it was developed in a pediatric cohort), emergency physicians should become familiar with scrotal ultrasonography (US), the imaging modality of choice for testicular pain [9-11]. While it is understandable that an emergency physician may not be able to expertly identify a whirlpool sign (the presence of a spiral-like pattern of the spermatic cord) [11] or calculate the elevated resistive index on Doppler blood flow, being able to decipher equal bilateral blood flow is imperative. Ultimately, in most malpractice cases, even if a missed diagnosis is attributed to an incorrect radiology read, the emergency physician pays the majority of the malpractice amount [12]. The easiest images to look at are the comparison or “buddy” images. Figures 1-2 show patient 1’s ultrasound images while Figures 3-4 show patient 2’s corresponding images. A side-by-side comparison shows that in the case of patient 1, the differences are relatively obvious. Figure 1 shows the heterogenous echotexture (signs of ischemia) of the right testicle as compared to the left, unlike Figure 3 in which both testicles have a similar homogenous echogenicity throughout. Figure 2 shows decreased/absent Doppler blood flow to the right testicle as compared to the left, unlike Figure 4 in which the Doppler images of both testicles look almost identical in the frequency and intensity of arterial and venous signals recorded. Again, in the case of patient 1, the free-standing emergency physician could have identified the torsion earlier than the official radiology read. Meaning, patient 1 could have been transferred to our center 60 minutes sooner than he was. Manual detorsion of the testis (Figure 5) could also have been attempted while awaiting the transfer. Patient 2’s ultrasound, on the other hand, was seemingly normal, meaning, he theoretically could have been discharged 90 minutes sooner than he was.

While some suggest the use of an algorithm to decide whether a scrotal ultrasound is necessary, with the widespread availability of US in EDs these days, the authors opine that all patients with acute scrotal pain who present to the ED should be evaluated with ultrasonography.

## Conclusions

In this report, we present the cases of two young men of similar age, with similar medical histories, who present to the emergency department with a similar history of the present illness. In both cases, the diagnostic evaluation was the same, however, the results were quite different. It is vital that emergency physicians are vigilant in pursuing the diagnosis of testicular torsion with a thorough physical exam and diagnostic testing. It is also important to implement methods that can expedite definitive care. In our case, both patients were lucky enough to have satisfactory outcomes at the time of discharge. However, we can see that in the case of patient 1 with testicular torsion, the operating room could have been reached 60 to 90 minutes earlier. Taking into account the potential delays that could have occurred, such as the patient delaying presentation to the emergency department, or the department having long wait times, or the delay in night shift radiology reads, or even the delays that tend to accompany transfers, 60 to 90 minutes could easily become the difference between viability and non-viability.
